# Editorial: Language Representation and Learning in Cognitive and Artificial Intelligence Systems

**DOI:** 10.3389/frobt.2020.00069

**Published:** 2020-06-16

**Authors:** Massimo Esposito, Giovanni Luca Masala, Bruno Golosio, Angelo Cangelosi

**Affiliations:** ^1^Institute for High Performance Computing and Networking of the National Research Council of Italy, Naples, Italy; ^2^Manchester Metropolitan University, Manchester, United Kingdom; ^3^University of Cagliari, Cagliari, Italy; ^4^The University of Manchester, Manchester, United Kingdom

**Keywords:** Natural Language Processing (NLP), artificial intelligence, cognitive systems, robotics, deep learning, machine learning, language representation and language processing

## Introduction

In recent years, the rise of deep learning has transformed the field of Natural Language Processing (NLP), thus, producing models based on neural networks with impressive achievements in various tasks, such as language modeling (Devlin et al., 2019), syntactic parsing (Pota et al., 2019), machine translation (Artetxe et al., 2017), sentiment analysis (Fu et al., 2019), and question answering (Zhang et al., 2019). This progress has been accompanied by a myriad of new end-to-end neural network architectures able to map input text to some output prediction. On the other hand, architectures inspired by human cognition have recently appeared (Dominey, 2013; Hinaut and Dominey, 2013; Golosio et al., 2015), this is aimed at modeling language comprehension and learning by means of neural models built according to current knowledge on how verbal information is stored and processed in the human brain.

Despite the success of deep learning in different NLP tasks and the interesting attempts of cognitive systems, natural language understanding still remains an open challenge for machines.

The goal of this Research Topic is to describe novel and very interesting theoretical studies, models, and case studies in the areas of NLP as well as Cognitive and Artificial Intelligence (AI) systems, based on knowledge and expertise coming from heterogeneous but complementary disciplines (machine/deep learning, robotics, neuroscience, psychology).

## Papers Included in This Research Topic

Stille et al. propose a large-scale neural model, including cognitive and lexical levels of the human neural system, with the aim of simulating the human behavior occurring in medical screenings. The large-scale neural model is biologically inspired and built by exploiting the Neural Engineering Framework and the Semantic Pointer Architecture. The authors simulate parts of both the screenings, using either the normal neural model or the neural model including neural deficits. The simulated screenings are focused on the detection of developmental problems in lexical storage and retrieval, as well as of mild cognitive impairment and early dementia.

Jacobs proposes a heuristic tool called SentiArt for realizing different sentiment analyses for text segments and figures. The tool uses vector space models together with theory-guided and empirically validated label lists to compute the valence of each word in a text by locating its position in a 2d emotion potential space spanned by the >2 million words of the vector space model. By means of two computational poetics studies, the author experimentally shows the ability of SentiArt to determine the emotion of text passages and to compute emotional figure profiles and personality figure profiles for main characters from the book series (stories, novels, plays, or ballads).

Ferrone and Zanzotto describe a survey aimed to deeply investigate the link between symbolic and distributed/distributional representations of Natural Language. In particular, the survey describes the general concept of representation, the notion of concatenative composition and the difference between local and distributed representations. Furthermore, it deeply addresses the general issue of compositionality, analyzing three different approaches: compositional distributional semantics, holographic reduced representations and recurrent neural networks.

Nakashima et al. presents a new unsupervised machine learning method for phoneme and word discovery from multiple speakers. Human infants can acquire knowledge of phonemes and words from interactions with their mother as well as with others surrounding them. Authors propose a phoneme and word discovery method that simultaneously uses non-parametric Bayesian double articulation analyzer and deep sparse autoencoder with parametric bias in a hidden layer. Their system reduces the negative effect of speaker-dependent acoustic features in an unsupervised manner by using a speaker index required to be obtained through another speaker recognition method. This can be regarded as a more natural computational model of phoneme and word discovery by humans, because it does not use transcription.

Wallbridge et al. proposes a dynamic method of communication between robots and humans in order to generate Spatial Referring Expressions describing a location. The focus of most algorithms for generation is to create a non-ambiguous description, but this is not how people naturally communicate. The authors call dynamic description how humans tend to give an underspecified description and then rely on a strategy of repair to reduce the number of possible locations or objects until the correct one is identified. The authors present a method for generating these dynamic descriptions for Human Robot Interaction, using machine learning to generate repair statements in a two-dimensional environment (game-like scenario).

Miyazawa et al. presents a unified framework, integrating a cognitive architecture in a real robot for the simultaneously comprehension of concepts, actions, and language. Their integration is based on various cognitive modules and leveraging mainly multimodal categorization by using multilayered multimodal latent Dirichlet allocation (mMLDA). The integration of reinforcement learning and mMLDA enables actions based on understanding. Furthermore, the mMLDA, in conjunction with grammar learning and based on the Bayesian hidden Markov model, allows the robot to verbalize its own actions and understand user utterances. Decision making and language understanding by using abstracted concepts are verified using a real robot.

## Conclusions

Despite relevant progress having been made in the field of AI applied to NLP in the last decade, the goal of creating truly human-like intelligent systems still seems very distant. The difficulties encountered in the development of the most recent systems clearly show that the problem of human-machine interaction through natural language can no longer be addressed as a simple input-output problem. To make a qualitative leap, AI systems should become more complete multimodal systems which are able to integrate skills in areas of AI that are currently treated separately and should be capable of developing an internal representation of the external world through the combination of other information besides the verbal one. Such combination can be achieved through the integration of AI systems in robots. Embodied architectures in robots should be able to learn in a similar way to humans through interaction with humans themselves and be capable of proactively adapting operating in the environment to find the information necessary to learn and to interact more profitably with humans. From this perspective, perhaps the approaches inspired by neuroscience and cognitive models can still provide new important ideas to this field.

## Author Contributions

All authors contributed equally to manuscript writing, read, and approved the final version.

### Conflict of Interest

The authors declare that the research was conducted in the absence of any commercial or financial relationships that could be construed as a potential conflict of interest.
